# Spi-B Promotes the Recruitment of Tumor-Associated Macrophages *via* Enhancing CCL4 Expression in Lung Cancer

**DOI:** 10.3389/fonc.2021.659131

**Published:** 2021-06-01

**Authors:** Qiumin Huang, Junrong Liu, Shuainan Wu, Xuexi Zhang, Zengtuan Xiao, Zhe Liu, Wei Du

**Affiliations:** ^1^ Department of Immunology, Biochemistry and Molecular Biology, 2011 Collaborative Innovation Center of Tianjin for Medical Epigenetics, Tianjin Key Laboratory of Medical Epigenetics, Tianjin Medical University, Tianjin, China; ^2^ Department of Lung Cancer Center, Tianjin Medical University Cancer Institute and Hospital, Tianjin, China; ^3^ Key Laboratory of Immune Microenvironment and Disease of the Ministry of Education, Tianjin Medical University, Tianjin, China; ^4^ Tianjin Key Laboratory of Radiation Medicine and Molecular Nuclear Medicine, Institute of Radiation Medicine, Tianjin, China

**Keywords:** tumor-associated macrophages, lung cancer, CCL4, CD163, Spi-B

## Abstract

Tumor immune escape plays a critical role in malignant tumor progression and leads to the failure of anticancer immunotherapy. Spi-B, a lymphocyte lineage-specific Ets transcription factor, participates in mesenchymal invasion and favors metastasis in human lung cancer. However, the mechanism through which Spi-B regulates the tumor immune environment has not been elucidated. In this study, we demonstrated that Spi-B enhanced the infiltration of tumor-associated macrophages (TAMs) in the tumor microenvironment using subcutaneous mouse models and clinical samples of human lung cancer. Spi-B overexpression increased the expression of TAM polarization- and recruitment-related genes, including *CCL4*. Moreover, deleting *CCL4* inhibited the ability of Spi-B promoting macrophage infiltration. These data suggest that Spi-B promotes the recruitment of TAMs to the tumor microenvironment *via* upregulating *CCL4* expression, which contributes to the progression of lung cancer.

## Introduction

Lung cancer, including non-small cell lung cancer (NSCLC) and small cell lung cancer (SCLC), is one of the most prevalent cancers and is the leading cause of cancer-related deaths worldwide (1). Despite developments in targeted therapies and immunotherapy, the 5-year survival rate of patients with metastatic lung cancer remains below 5% (2, 3). Estimates suggest that the diagnosis and treatment of lung cancer patients at an early stage leads to improved prognosis (2). The mechanisms of lung carcinogenesis and the role of the microenvironment in tumor initiation remain to be elucidated. Understanding these mechanisms may pave the way for new treatments, especially personalized immunotherapy, which will reduce the mortality rate of lung cancer patients.

Carcinogenesis is a complicated multistage process that is influenced by tumor-intrinsic factors, the tumor microenvironment (TME), and extrinsic carcinogens. The TME is composed of the extracellular matrix, stromal cells, immune cells, and secreted proteins (4). Tumor-associated macrophages (TAMs) are a major component of the immunosuppressive TME and are derived from circulating monocytes (5). Macrophages are recruited to the TME by multiple chemotactic cytokines, including CCL2 (MCP-1), CCL3 (MIP-1α), CCL4 (MIP-1β), CCL5 (RANTES), and colony-stimulating factors (6–10). Most of these chemotactic cytokines are produced by tumor cells, fibroblasts, endothelial cells, and even TAMs themselves (11, 12). Macrophages are classified into M1 and M2 subtypes depending on whether the immune response is induced by Th1 or Th2 cells. M2 macrophages are further subdivided into M2a, M2b, and M2c cells based on their secreted cytokines and immune functions. When exposed to interleukin (IL) 4 produced by CD4^+^ T cells and other immunosuppressive stimuli in the TME, TAMs mostly present as the M2 phenotype (13, 14). TAMs release various cytokines into the TME to enhance tumor development and participate in multiple biological processes, including angiogenesis, cell invasion, cell migration, and tumor metastasis. High TAM infiltration is associated with poor prognosis in several solid tumors, including lung cancer (15).

Spi-B, an Ets transcription factor, plays an important role in the differentiation of B cells, plasmacytoid dendritic cells, and intestinal microfold cells (16–18). Recently, Spi-B has been detected in various malignant solid tumors (19–21), such as lung cancer, where it promotes mesenchymal invasion and autophagy-mediated anoikis resistance, thereby linking epithelial cancer metastasis with a lymphatic transcriptional program (20, 21). The present study aimed to investigate the role of Spi-B in the immune microenvironment of lung cancer. Our study showed that Spib enhanced TAM infiltration in subcutaneous mouse models. Further, Spi-B expression in tumor cells was significantly associated with CD163 in human TAMs, suggesting poor clinical outcomes. Transwell assays further revealed that Spi-B promoted TAM recruitment, possibly by enhancing *CCL4* expression. Our results demonstrate that Spi-B might be an important diagnostic biomarker for monitoring patients with lung cancer.

## Materials and Methods

### Experimental Animals

Female C57BL/6 mice (6 to 8 weeks old) were purchased from Beijing Vital River Laboratory Animal Technology. All animal experiments were performed accordance to guidelines approved by the animal ethics committee of Tianjin Medical University.

### Cell Lines

A549, H1299, HEK293T, and LLC1 cells were obtained from the American Type Culture Collection (ATCC, Manassas, VA, USA). CMT167 and RAW264.7 cells were purchased from Shanghai Zhong Qiao Xin Zhou Biotechnology Co., Ltd. (China). All cell lines were maintained in the recommended culture medium supplemented with 10% fetal bovine serum (FBS), 100 U penicillin/mL, and 100 mg streptomycin/mL in 5% CO_2_ at 37°C.

### Lentiviral Production and Transfection

The primers used for *Spib* cDNA amplification and the shRNA sequences targeting *Spib* and *Ccl4* are listed in **Table S1**. The plasmids were co-transfected into HEK293T cells along with the helper plasmids pMD2.BSBG, pMDLg/pRRE, and pRSV-REV using Polyethylenimine (PEI). Viral supernatants were harvested 24 h and 48 h after transfection. For infection, the target cell medium was replaced with virus-containing supernatant supplemented with 8 μg/mL polybrene (Sigma, 107689) and incubated for 8 h. This procedure was repeated daily for three consecutive days to establish stable cell lines.

### Subcutaneous Mouse Models and Immune Cell Subset Analysis

A total of 5 × 10^5^ LLC1 cells expressing empty vector or *Spib* were subcutaneously injected into C57BL/6 mice on the right back flank. After 14 days, *in situ* tumors were harvested for immune cell subset analysis. The tumors were minced into small pieces and digested in DMEM supplemented with collagenase I (5 μg/mL), hyaluronidase (5 μg/mL), and deoxyribonuclease I (5 μg/mL) for 30 min at 37°C with gentle agitation. Single-cell suspensions were obtained by gently passing the enzyme-treated tumor tissue through a 70-µm cell strainer. The cell suspensions were subjected to sequential Percoll (Sigma) gradient centrifugation and resuspended in PBS supplemented with 1% FBS for downstream flow cytometry analysis. For *in vivo* metastasis assays, the mice were sacrificed under deep anesthesia at 6 weeks after subcutaneous injection and metastatic nodules in the lungs was counted and assessed by histology using Hematoxylin and Eosin (HE). For survival assays, the mice were inspected every day for health issues, and deaths were recorded for three months after resection.

A total of 1 × 10^6^ CMT167 cells transduced with lentivirus-expressing shRNA against *Spib* or control vector were subcutaneously injected into C57BL/6 mice on the right back flank. After 14 days, *in situ* tumors were excised to analyze tumor growth.

### Flow Cytometry Analysis

Single-cell suspensions from mouse subcutaneous tumors were prepared as described above, counted, and resuspended in PBS at a concentration of 1×10^7^ live cells/mL. One hundred microliters of each sample was plated for cell staining. Surface staining was performed at room temperature for 30 min, and intracellular staining was performed using a Foxp3-transcription factor staining kit (eBioscience). Live/dead cell discrimination was performed using Fixable Viability Stain 520 (BD Biosciences). Anti-mouse antibodies against the following antigens were used for flow cytometry: CD3 PerCP-CY5.5 (17A2, BioLegend, 1:100), CD45 PerCP (30-F11, BioLegend, 1:200), NK1.1 APC (PK136, BD Biosciences, 1:50), Foxp3 PE (MF23, BD Biosciences, 1:100), Ly6G PE (1A8, BioLegend, 1:100), Ly6C APC (HK1.4, BioLegend, 1:100), CD11b BV421 (M1/70, BioLegend, 1:100), CD11b PerCP-CY5.5 (HL3, BioLegend, 1:100), F4/80 APC (T45–2342, BD Biosciences, 1:50), CD25APC (3C7, BioLegend, 1:100), CD4 APC (GK1.5, BioLegend, 1:100), and CD8a PE (53-6.7; BioLegend, 1:100). All flow cytometry analyses were performed using a Fortessa flow cytometer (BD Biosciences) and analyzed using FlowJo software (TreeStar).

### Real-Time Quantitative PCR (qRT-PCR) and Gene Expression

Total RNA was extracted from cell lines using TRIzol reagent (Invitrogen) and reverse transcribed into cDNA (Thermo Scientific, USA). The synthesized cDNA was assayed using SYBR Green PCR kits (DBI Bioscience) on an ABI 7900 Real-Time PCR system. Relative fold changes were normalized to GAPDH expression using the 2^−ΔΔCt^ method. The primers used for PCR are listed in **Table S1**.

### Western Blotting

Cells were lysed in RIPA buffer and total protein was quantified using the bicinchoninic acid method. Then, proteins were loaded and resolved using sodium dodecyl sulfate-polyacrylamide gel electrophoresis and transferred onto a nitrocellulose membrane. The membranes were immunoblotted with antibodies against the following antigens: Spi-B (Novus, NBP2-37458, 1:1000) and β-actin (Sigma, MAB1501, 1:5000). Immunoreactive bands were detected using enhanced chemiluminescence detection substrate (Millipore).

### T Cell Function Assays

Spleens from naive mice were isolated and passed through 70-micrometer filters to generate single-cell suspensions. After red blood cell lysis, T cells were purified using negative selection with magnetic beads using the EasySep™ mouse T cell isolation kit, according to the manufacturer’s instructions. CD11b^+^ myeloid cells from subcutaneous tumors derived from LLC1 cells expressing *Spib* or empty vector were purified using anti-CD11b^+^ microbeads. T cells were plated in 96 U-bottom wells (2.5×10^4^ cells per well) coated with 1 μg/mL anti-CD3 and 5 μg/mL anti-CD28 antibodies. Then, T cells were co-cultured with CD11b^+^ myeloid cells in a 3:1 T cell:myeloid cell ratio at 37°C for 48 h. For cytokine staining, we used a GolgiStop Fixation/Permeabilization kit (BD Biosciences). For T cell proliferation assays, carboxyfluorescein succinimidyl ester (CFSE, Invitrogen) was used to stain T cells. CFSE-labeled CD8^+^ T cells were co-cultured with myeloid cells in a 3:1 ratio. After 48 h, the cells were harvested, and CFSE signal in the CD8^+^ T cells was measured by flow cytometry.

### Histological Staining

Excised mouse lungs, mouse subcutaneous tumors and human lung tumors were fixed with 4% formaldehyde for 24 h and embedded in paraffin for slide preparation. Tissue sections were stained with hematoxylin and eosin (H&E) using standard reagents and protocols. For immunohistochemical analysis, tissue sections were immunostained with antibodies against Spi-B (Novus, NBP2-37458, 1:100), CD163 (Abcam, ab182422, 1:100), and CD8 (Cell Signaling Technology, 98941S, 1:100), and processed according to standard DAB staining protocols, as described previously (20).

### Recruitment Assays

RAW264.7 macrophage migration was examined in conditioned medium without 10% FBS. A total of 1 × 10^5^ RAW264.7 cells were seeded into the upper chamber of 24-well transwell inserts (Corning). Conditioned medium was added to the lower chambers. RAW264.7 cells were incubated for 5 h in 5% CO_2_ at 37°C. The macrophages that migrated and attached to the lower surface of the transwell membrane were fixed with 4% paraformaldehyde and stained with 0.5% crystal violet. Cells in five random fields were counted using a phase-contrast microscope (200× magnification) to analyze the macrophage migration rate.

### Bioinformatics Analysis

The mRNA microarray data was obtained from the GEO database (GEO database access no. GSE90645) (20). Gene expression profiles (.gct files) were used as the input for gene set enrichment analysis (GSEA; version 4.0.2). GSEA was run using the gene-ranking metric Signal2 noise with C5 MsigDbcollection. A total of 1,000 permutations were used to calculate p values. A nominal p < 0.01 was used as the cut-off criterion for determining significant enrichment scores.

TIMER is a comprehensive online resource for studying immune infiltration in various cancer types. We used TIMER on lung cancer sample data to analyze the correlation between Spi-B and key genes involved in macrophage polarization and recruitment.

### Statistical Analysis

GraphPad Prism and SPSS 18.0 were used for statistical analysis. The survival rates were determined with Kaplan-Meier analysis using Mantel-Cox log-rank testing. Statistical analysis for the comparison of two groups was performed using unpaired Student’s t-test. The results are shown as the mean ± standard deviation. P < 0.05 was considered statistically significant for all tests.

## Results

### *Spib* Overexpression Increases TAM Infiltration and Promotes Lung Cancer Progression in Subcutaneous Mouse Models

**Figure 1:**
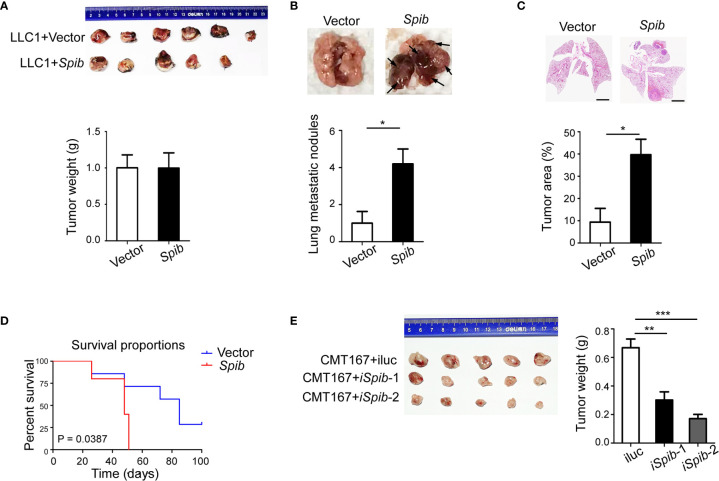
*Spib* overexpression promotes lung cancer progression in subcutaneous mouse models. **(A)** Subcutaneous tumors *in situ* were excised 2 weeks after inoculation with LLC1 cells expressing empty vector or *Spib*. Upper panel, images of subcutaneous tumors. Lower panel, tumor weight. **(B)** The mice were sacrificed and lung metastases were analyzed 6 weeks after inoculation with LLC1 cells expressing empty vector or *Spib* (n = 5). Upper panel, representative images of lungs. Black arrow, metastatic nodules. Lower panel, quantitation of lung metastatic nodules. **(C)** H&E staining of lungs from mice subcutaneously injected with vector or *Spib*-expressing LLC1 cells (n = 5). Scale bars, 2 mm. Upper panel, representative H&E staining in lungs. Lower panel, the tumor burden was measured as the percentage of the tumor area versus the total lung area. **(D)** Kaplan-Meier survival curve of mice subcutaneously injected with vector or *Spib*-expressing LLC1 cells. Survival between LLC1 cells expressing empty vector and LLC1 cells expressing *Spib* was significantly different (P < 0.05; n = 6, vector; and n = 5, *Spib*). **(E)** CMT167 cells expressing control or shRNA against *Spib* were subcutaneously injected into C57BL/6 mice. Two weeks later, subcutaneous tumors were removed. Left, images of subcutaneous tumors. Right, tumor weight. Mean ± SD. *P < 0.05. **P < 0.01. ***P < 0.001.

To investigate the role of Spi-B in the lung carcinoma immune microenvironment, we used a mouse subcutaneous engraftment model and *Spib*-expressing LLC1 cells (**Figure S1A**). *Spib* expression had a modest effect on LLC1-derived subcutaneous tumor growth (**Figure 1A**, P > 0.05). At 6 weeks after injection, subcutaneous engraftment of *Spib*-expressing LLC1 cells showed significantly more lung metastatic nodules and relative tumor area than vector-transduced cells (**Figures 1B, C**). No metastases were found in other organs. We also monitored the survival of subcutaneous tumor-bearing mice. The survival of mice injected with *Spib*-expressing cells was significantly reduced compared with vector-transduced cells in subcutaneous mouse models (**Figure 1D**). Conversely, the knockdown of *Spib* in CMT167 cells, a murine lung cancer cell line that expresses endogenous *Spib* (**Figures S1B, C**), showed a significant reduction in CMT167-derived subcutaneous tumor growth (**Figure 1E**). These data indicate that *Spib* expression promotes lung cancer progression in subcutaneous mouse models.

**Figure 2:**
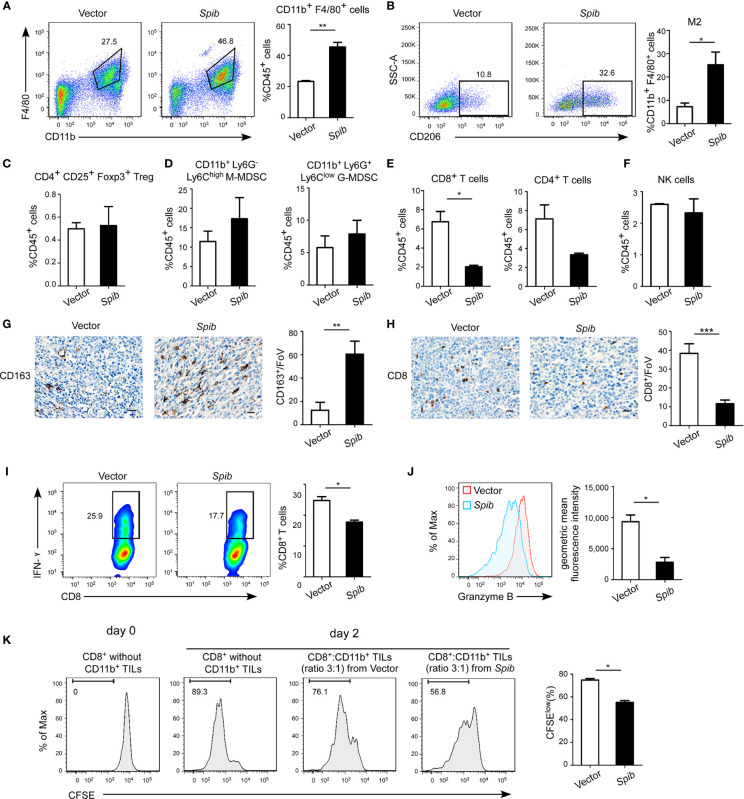
*Spib* overexpression increases TAM infiltration in subcutaneous mouse models. **(A)** Representative flow cytometry profiles and percentages of TAMs in CD45^+^ TILs in subcutaneous tumors. n = 3-4. **(B)** Representative flow cytometry profiles and percentages of CD206^+^ cells in CD11b^+^ F4/80^+^ cell populations in subcutaneous tumors. n = 3. **(C)** Quantification of Tregs in CD45^+^ TILs in subcutaneous tumors by flow cytometry. n = 3-4. **(D)** Quantification of monocytic myeloid-derived suppressor cells (M-MDSCs) and granulocytic myeloid-derived suppressor cells (G-MDSCs) in CD45^+^ TILs in subcutaneous tumors by flow cytometry. n = 3-4. **(E)** Quantification of CD8^+^ T cells and CD4^+^ T cells in CD45^+^ TILs in subcutaneous tumors by flow cytometry. n = 3-4. **(F)** Quantification of NK cells in CD45^+^ TILs in subcutaneous tumors by flow cytometry. n = 3-4. **(G)** Immunostaining with anti-CD163 was performed in subcutaneous tumors derived from LLC1 cells expressing *Spib* or empty vector. Scale bars, 20 µm. Left, representative pictures of CD163 staining. Right, quantified CD163 expression in subcutaneous tumors. FoV = field of view. n = 3 mice for each group. **(H)** Immunostaining with anti-CD8 was performed in subcutaneous tumors derived from LLC1 cells expressing *Spib* or empty vector. Scale bars, 20 µm. Left, representative pictures of CD8 staining. Right, quantified CD8 expression in subcutaneous tumors. n = 3 mice for each group. **(I, J)** CD11b^+^ cells purified from subcutaneous tumors derived from LLC1 cells expressing *Spib* or empty vector were cocultured with T cells for 2 days. Quantification of IFN-γ^+^ in CD8^+^ T cells by flow cytometry **(I)**. Quantification of the geometric mean fluorescence intensity of granzyme B in CD8^+^ T cells **(J)**. n= 3. **(K)** *In vitro* suppressive activity of tumor-infiltrating CD11b^+^ cells purified from subcutaneous tumors derived from LLC1 cells expressing *Spib* or empty vector. Representative histograms of CD8^+^ T cell proliferation (left panel). Quantification of CD8^+^ T cell proliferation using carboxyfluorescein succinimidyl ester (CFSE) (right panel). n = 3. Mean ± SD. *P < 0.05. **P < 0.01. ***P < 0.001.

Flow cytometry analysis of TAMs, regulatory T cells (Tregs), and myeloid-derived suppressor cells (MDSCs) with immune-suppressive capabilities isolated from subcutaneous tumors indicated that the percentages of total F4/80^+^ CD11b^+^ TAMs in CD45^+^ tumor-infiltrating leukocytes (TILs) and CD206^+^ F4/80^+^ CD11b^+^ macrophages (M2 macrophages) in total TAMs were both markedly increased in *Spib*-expressing tumors (**Figures 2A, B** and **S2A**). However, no differences in the Treg and MDSC percentages were observed between *Spib*-expressing tumors and control tumors (**Figures 2C, D** and **S2B, C**). The proportion of CD8^+^ T cells was significantly reduced in *Spib*-expressing tumors compared with control tumors (**Figures 2E** and **S2D**), indicating a decreased cytotoxic anti-tumor immune response. We did not observe a significant change in the percentage of CD4^+^ T cells or natural killer (NK) cells (**Figures 2E, F** and **S2D, E**). Staining with CD163, a marker of M2 macrophages, confirmed a significant increase in the percentage of M2 macrophages in *Spib*-expressing subcutaneous tumors and lung metastases (**Figures 2G** and **S3**). Moreover, CD8 immunostaining in subcutaneous tumors confirmed a marked reduction in the percentage of CD8^+^ T cells in *Spib*-expressing tumors compared to control tumors (**Figure 2H**). To identify the role of TAMs from *Spib*-expressing tumors in T cell responses, CD11b^+^ cells were isolated from subcutaneous tumors and incubated with CD8^+^ T cells from the spleen of C57BL/6 mice for two days. Myeloid cells from *Spib*-expressing tumors significantly inhibited interferon (IFN)-γ and granzyme B expression in CD8^+^ T cells compared with myeloid cells from control tumors (**Figures 2I, J**). Furthermore, myeloid cells from *Spib*-expressing tumors suppressed CD8^+^ T cell proliferation to a greater extent than myeloid cells from control tumors (**Figure 2K**), indicating that myeloid cells from Spib-expressing tumors exhibit more suppressive activity. Collectively, these results suggest that *Spib* overexpression in subcutaneous mouse models increases the infiltration of TAMs, especially M2 macrophages, and promotes lung cancer progression.

### Spi-B Expression in Human Lung Cancer Tissues is Positively Correlated with CD163 Expression and Predicts Poor Survival of NSCLC Patients

**Figure 3:**
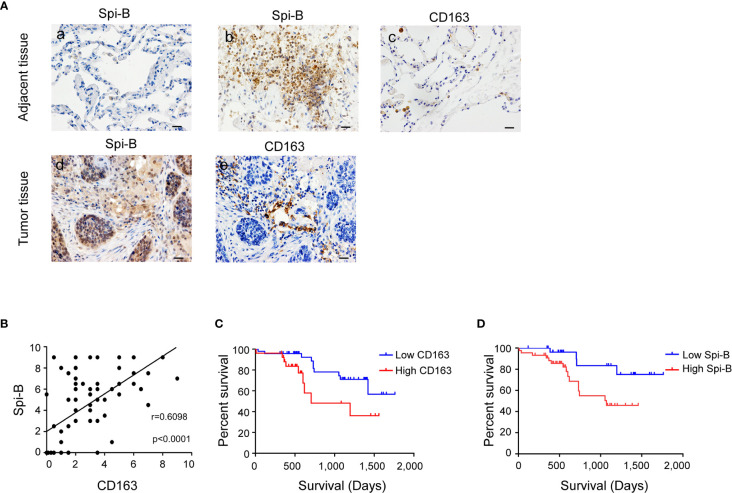
Spi-B expression positively correlates with the proportion of CD163^+^ macrophages in human lung cancer tissues. **(A)** Immunostaining of human CD163 and Spi-B in paraffin-embedded lung tissues using phase-contrast microscopy (400×). Representative images show a lack of Spi-B staining in normal lung epithelial cells (a), but positive Spi-B staining in lymphocytes (b) and positive CD163 staining in alveolar macrophages (c). In lung cancer tissues, CD163-expressing TAMs surrounded and infiltrated cancer masses of strong Spi-B staining (d, e). Scale bars, 20 µm. **(B)** Semiquantitative scoring was performed. CD163 scores were associated with Spi-B scores (r = 0.6098, P < 0.0001). **(C)** Kaplan-Meier survival rates for 73 subjects with low (staining scores ≤ 3, n = 47, blue line) versus high (staining scores > 3, n = 26, red line) CD163 expression were compared (P = 0.0307). **(D)** Kaplan-Meier survival rates for 73 subjects with low (staining scores < 4, n = 29, blue line) versus high (staining scores ≥ 4, n = 44, red line) Spi-B expression were compared (P = 0.0366).

To determine whether Spi-B expression promotes TAM infiltration in human lung cancer tissues, we examined the expression of CD163 and Spi-B in tissue samples obtained from 79 NSCLC patients admitted to the Tianjin Medical University Cancer Institute and Hospital. Epithelial cells in normal lung tissues did not express Spi-B (**Figure 3A**, a). In tumor-adjacent tissues, lymphocytes expressed Spi-B (**Figure 3A**, b), and alveolar macrophages expressed CD163 (**Figure 3A**, c). In human lung cancer tissues, TAMs expressing CD163 surrounded and infiltrated the cancer mass of strong Spi-B staining (**Figures 3A**, d, e). When immunostained tissue sections were quantified, a highly significant positive correlation was found between Spi-B^+^ cells in lung cancers and CD163^+^ macrophages (**Figure 3B**). We also used TIMER to analyze the relationship between Spi-B and CD163 expression. The results revealed that Spi-B expression was positively associated with CD163 expression in adenocarcinoma (ADC, **Figure S4A**) and squamous cell carcinoma (SCC, **Figure S4B**).

Next, we investigated the prognostic impact of CD163^+^ macrophages in patients with NSCLC. Patients with high CD163^+^ macrophage density had poorer overall survival (OS) than patients with low CD163^+^ macrophage density (**Figure 3C**). We also identified that patients whose tumors showed high Spi-B staining intensity had poorer OS than those whose tumors showed low staining intensity (**Figure 3D**). These results suggest that CD163^+^ macrophages may serve as a significant prognostic factor for NSCLC.

### Spi-B Promotes Recruitment of Macrophages

**Figure 4:**
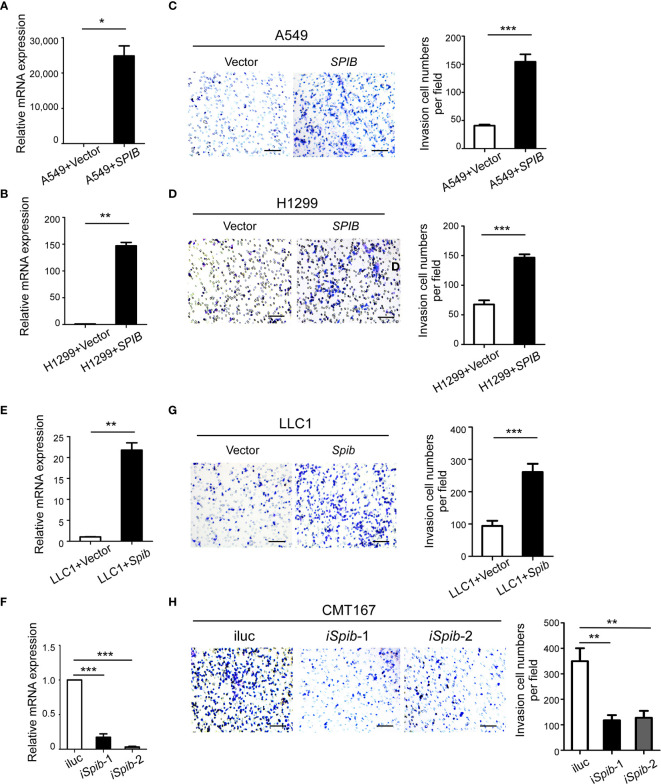
*SPIB* promotes macrophage recruitment. **(A, B, E, F)** Human *SPIB* overexpression efficiency in A549 **(A)** and H1299 **(B)** cells verified by qRT-PCR. Murine *Spib* overexpression efficiency in LLC1 cells **(E)** and *Spib* silencing efficiency in CMT167 cells **(F)** verified by qRT-PCR. *P < 0.05. **P < 0.01. ***P < 0.001. **(C, D, G, H)** Recruitment assay. Left, representative transwell images of recruited macrophages. Right, relative fold changes of recruited macrophages in each image field observed by phase-contrast microscopy (200×). Columns, mean (n = 3). Bars, SD. **P < 0.01. ***P < 0.001. Scale bars, 100 µm.

To assess the effect of Spi-B on macrophage infiltration during lung cancer *in vitro*, human lung cancer cells (A549 and H1299), which do not express endogenous Spi-B, were transfected with *SPIB*. Transfection was verified by qRT-PCR (**Figures 4A, B**). A transwell assay was performed to detect the effect of Spi-B on macrophage recruitment. As shown in **Figures 4C, D**, we found significantly increased recruitment of macrophages in Spi-B-expressing lung cancer cells compared with control cells. Then, LLC1 cells were transfected with the murine *Spib* gene (**Figure 4E**), and CMT167 cells were transfected with shRNAs to knockdown *Spib* (**Figure 4F**). We observed that *Spib* overexpression in LLC1 cells exhibited similar effects on macrophage recruitment (**Figure 4G**). In contrast to overexpression, *Spib* knockdown significantly inhibited macrophage chemotaxis compared with CMT167-shRNA-scramble cells (**Figure 4H**), supporting the conclusion that Spi-B expression in tumor cells promotes TAM recruitment.

### Spi-B Upregulates the Expression of TAM Polarization- and Recruitment-Related Genes

**Figure 5:**
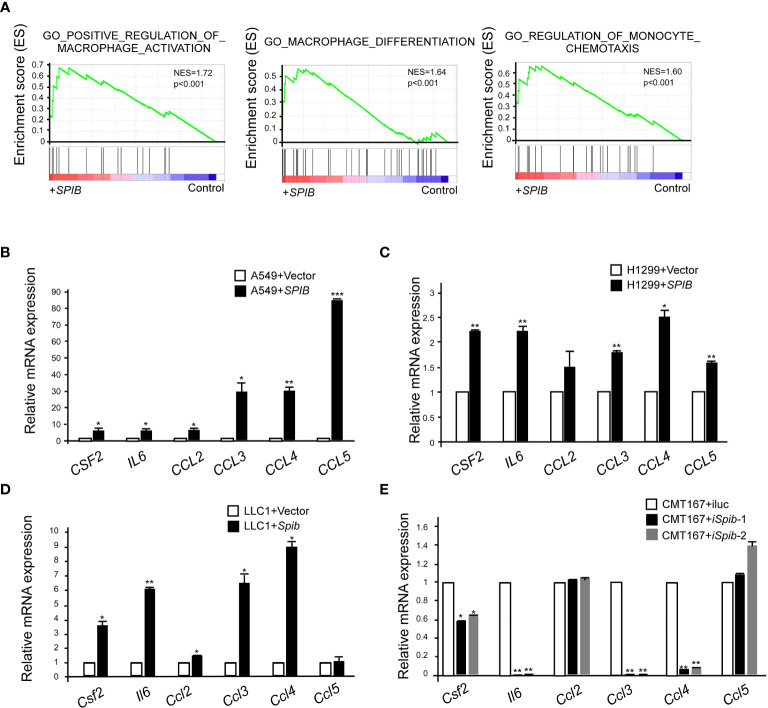
*SPIB* upregulates the expression of TAM polarization- and recruitment-related genes. **(A)** GSEA of C5 signatures (positive regulation of macrophage activation, macrophage differentiation, and regulation of monocyte chemotaxis) in microarray data of A549 cells expressing *SPIB* and control vector. **(B–E)** Validation of microarray data by RT-qPCR. RNA was purified from A549 cells expressing *SPIB* or empty vector **(B)**, H1299 cells expressing *SPIB* or empty vector **(C)**, LLC1 cells expressing *Spib* or empty vector **(D)**, and CMT167 cells with knockdown of *Spib* or control **(E)**. Mean ± SD. *P < 0.05. **P < 0.01 ***P < 0.001.

To better understand the molecular mechanisms underlying Spi-B-mediated TAM recruitment, we performed a GSEA of microarray data from Spi-B-expressing A549 cells and control cells. We found that Spi-B overexpression significantly enriched the positive regulation of macrophage activation, macrophage differentiation, and regulation of monocyte chemotaxis (NES=1.72, NES=1.64, and NES=1.60, respectively) (**Figure 5A**). Next, we performed quantitative RT-PCR to confirm Spi-B-dependent differential transcription of cytokines and chemokines involved in TAM polarization and macrophage recruitment. We examined CSF2, which promotes macrophage differentiation; IL6, which is involved in TAM polarization; and the chemokines, CCL2, CCL3, CCL4, and CCL5, which are associated with macrophage infiltration. *CSF2*, *IL6*, *CCL3*, *CCL4*, and *CCL5* mRNA expression was significantly upregulated, while *CCL2* mRNA expression was slightly upregulated in Spi-B-expressing A549 and H1299 cells compared with control cells (**Figures 5B, C**). We also performed qRT-PCR in murine lung cancer cells to confirm these results. Consistently, *Spib* knockdown in CMT167 cells caused reciprocal changes in the expression of *Csf2*, *Il6*, *Ccl3*, and *Ccl4* compared with *Spib* expression in LLC1 cells (**Figures 5D, E**). However, the expression of *Ccl2* and *Ccl5* was moderately altered in CMT167 cells transfected with *Spib*-shRNA and LLC1 cells transfected with *Spib* (**Figures 5D, E**). These data indicate that Spi-B regulates TAM polarization- and recruitment-related gene expression.

### Spi-B Enhances Macrophage Recruitment by Upregulating the Expression of *CCL4*

**Figure 6:**
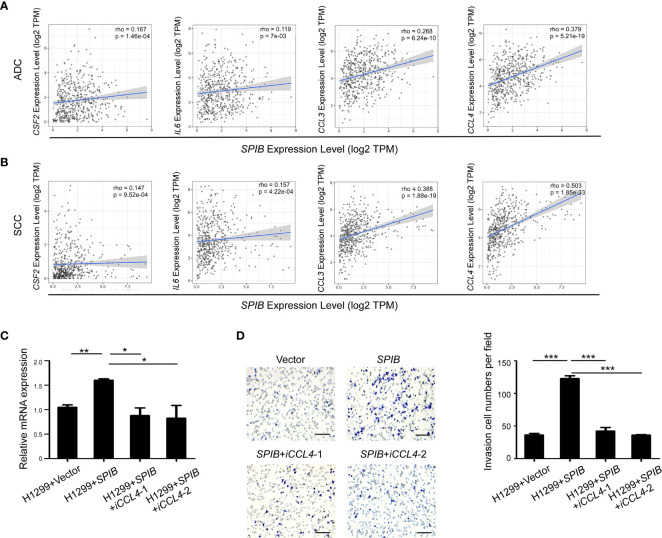
*CCL4* knockdown inhibits Spi-B-induced macrophage recruitment. **(A**, **B)** The correlation between the expression of *SPIB* and *CSF2, IL6, CCL3* and *CCL4* are shown in ADC **(A)** and SCC **(B)**. **(C)** *CCL4* silencing efficiency in H1299 cells expressing *SPIB* verified by RT-qPCR. Mean ± SD. *P < 0.05. **P < 0.01. **(D)** Recruitment assay in H1299 cells co-transfected with *SPIB* and *CCL4*-ShRNA. Left, representative transwell images of the recruited macrophages. Right, relative fold changes of recruited macrophages in each image field (200×). Mean ± SD. ***P < 0.001. Scale bars, 100 µm.

We used TIMER to further validate the correlation between the expression of Spi-B and TAM polarization- and recruitment-related genes in lung cancer tissues. The results showed that *SPIB* expression was positively associated with *CSF2*, *IL6*, *CCL3*, and *CCL4* expression in ADC and SCC (**Figures 6A, B**). Moreover, the expression of *CCL4* was the most positively correlated with *SPIB* expression. Next, we repressed *CCL4* expression in *SPIB*-overexpressing H1299 cells. The expression of *CCL4* was downregulated in H1299 cells co-transfected with *SPIB* and *CCL4*-ShRNA (**Figure 6C**). As expected, *CCL4* repression completely abolished Spi-B-induced macrophage recruitment (**Figure 6D**). These results suggest that Spi-B promotes TAM recruitment *via CCL4* upregulation.

## Discussion

The interaction between cancer cells and the TME is crucial for tumor progression and metastasis. Here, we identified that the ectopic expression of the lymphocyte-restricted transcription factor, Spi-B, in lung cancer cells promotes the recruitment of TAMs to the TME. Physiologically, Spi-B is expressed in B cells, plasmacytoid dendritic cells, T cell progenitors, and intestinal microfold cells (16–18, 22). Deleting Spi-B decreases B-cell activation and T cell-dependent humoral immune responses, indicating that Spi-B is required for sustaining germinal centers and memory B cell differentiation (16, 23). In addition, Spi-B is required for the development of human plasmacytoid dendritic cells and intestinal M cells with antigen presentation capacity. Based on the function of Spi-B during immune cell development, we investigated the role of Spi-B expression in lung cancer cells in regulating the composition of immune cells in the TME.

We found that Spi-B expressing promotes lung cancer metastasis in a subcutaneous mouse model. In this study, we analyzed immune cell infiltration into subcutaneous tumors using flow cytometry. The percentage of TAMs was markedly increased in *Spib*-expressing tumors compared with control tumors, while the percentage of CD8^+^ T cells was significantly reduced. Using immunostaining, we observed that CD163^+^ TAMs closely surrounded Spi-B-positive tumor cells in human lung cancer tissues. Furthermore, transwell assays showed that Spi-B overexpression in lung cancer cells enhanced macrophage migration. In contrast, silencing Spi-B reduced the number of migrating macrophages. These findings suggest that Spi-B expression in lung cancer cells is associated with macrophage infiltration.

By analyzing microarray data from Spi-B-expressing A549 cells and control cells, we found that the expression of macrophage polarization- and recruitment-related genes, including *CSF2*, *IL6*, *CCL3*, and *CCL4*, was significantly upregulated by Spi-B. CSF2 is an important survival, proliferation, and differentiation factor for macrophages and neutrophils, and it promotes the infiltration of monocytes and macrophages into injured tissues (24–26). CSF2 also regulates the switch from the proinflammatory M1 phenotype to the M2 phenotype *via* the p-Stat5 pathway (24, 26, 27). IL6, a proinflammatory cytokine secreted by various cell types, plays several biological roles in different cells (28). In response to specific microbial molecules, IL6 can be produced by macrophages involved in the host immune defense (28). In the TME, IL6 also regulates immune cell transformation from monocytes to a suppressive M2 phenotype, thereby promoting the proliferation and metastasis of various cancers, including lung (29), breast (30), hepatocellular (31), prostate (32), colorectal (33), renal cell (34), cervical (35), and ovarian cancers (36). Thus, IL6 is an effective marker for predicting poor prognosis in cancer patients. The β-chemokines, MIP-1α (CCL3) and MIP-1β (CCL4), can induce macrophage recruitment through CCR5 (37), which is highly expressed on macrophages (38). In our study, *CSF2*, *IL6*, *CCL3*, and *CCL4* expression was upregulated by Spi-B overexpression and downregulated by Spi-B knockdown, suggesting *CSF2*, *IL6*, *CCL3*, and *CCL4* as the target genes of Spi-B. Correlation analysis using the TIMER database revealed that the expression of *CCL4* was the most positively associated with *SPIB* expression. Furthermore, knockdown of *CCL4* in Spi-B-overexpressing cells inhibited macrophage recruitment. These results indicate that Spi-B overexpression promotes macrophage infiltration by upregulating *CCL4* expression.

In the TME, M2 macrophages contribute to tumor progression by producing anti-inflammatory cytokines, while M1 macrophages play an important role in antitumor and proinflammatory activity by producing proinflammatory cytokines and reactive oxygen/nitrogen species (39). Spi-B overexpression promotes lung cancer cell metastasis, which is mostly associated with M2 macrophage recruitment. A previous study showed that *CCL4* expression was positively correlated with the infiltration of M2 macrophages (40, 41). Inhibiting CCR5 (a receptors of CCL4) can induce TAM repolarization, which is mediated *via* the STAT3/SOCS3 pathway in macrophages (42). CCR5-deficient mice exhibit reduced tumor formation compared with wild-type mice (43). These studies suggest that Spi-B enhances macrophage infiltration and contributes to M2 macrophage polarization *via* the CCL4-CCR5 axis to promote tumor progression.

In summary, the current study demonstrates that Spi-B-positive lung cancer cells play an important role in increasing TAM recruitment by upregulating the expression of various cytokines, such as CSF2, IL6, CCL3, and CCL4. TAMs in the TME promote tumor metastasis. Monitoring Spi-B expression and targeting the CCL4-CCR5 axis raises the possibility of precise diagnosis and treatment of lung cancer.

## Data Availability Statement

The data sets presented in this study can be found in online repositories. The names of the repository/repositories and accession number(s) can be found in the article/**Supplementary Material**.

## Ethics Statement

The studies involving human participants were reviewed and approved by the ethics committee of Tianjin Medical University Cancer Institute and Hospital. The patients/participants provided their written informed consent to participate in this study. The animal study was reviewed and approved by the animal ethics committee of Tianjin Medical University.

## Author Contributions

WD and ZL designed the study, wrote the manuscript, prepared figures and tables, and approved the final draft. WD, QH, JL, SW, and XZ performed the experiments. QH, JL, ZL, and WD analyzed and interpreted the data. ZX collected human lung cancer specimens, patient information, and patient consent. All authors contributed to the article and approved the submitted version.

## Funding

This work was supported by the National Natural Science Foundation of China (grants 81825017 and 81773034 to ZL, 81872319 to WD), the Ministry of Science and Technology of China (grant 2018YFC1313002 to ZL), and the Tianjin Municipal Science and Technology Commission (20JCZDJC00110 to ZL).

## Conflict of Interest

The authors declare that the research was conducted in the absence of any commercial or financial relationships that could be construed as a potential conflict of interest.
