# Supplementary material for: Spi-B Promotes the Recruitment of Tumor-Associated Macrophages via Enhancing CCL4 Expression in Lung Cancer
Source: Front Oncol. 2021 Jun 1;11:659131. doi: 10.3389/fonc.2021.659131 (PMC8205110; doi:10.3389/fonc.2021.659131)
Supplement: Supplementary file 1 [file DataSheet_1.pdf]

Spi-B promotes the recruitment of tumor-associated macrophages via enhancing CCL4 expression in lung cancer

Qiumin Huang, Junrong Liu, Shuainan Wu, Xuexi Zhang, Zengtuan Xiao, Zhe Liu, Wei Du

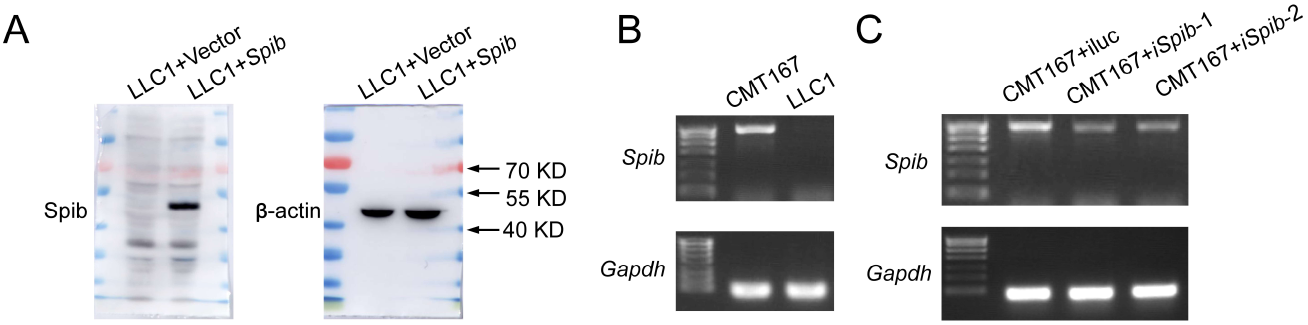

FIGURE S1 | The efficiency of *Spib* overexpression and knockdown, Related to FIGURE 1. (A) Immunoblots showed the efficiency of *Spib* overexpression. (B) RT-PCR showed transcription of *Spib* and *Gapdh* in CMT167 and LLC1 cell lines. (C) RT-PCR showed the efficiency of *Spib* knockdown.

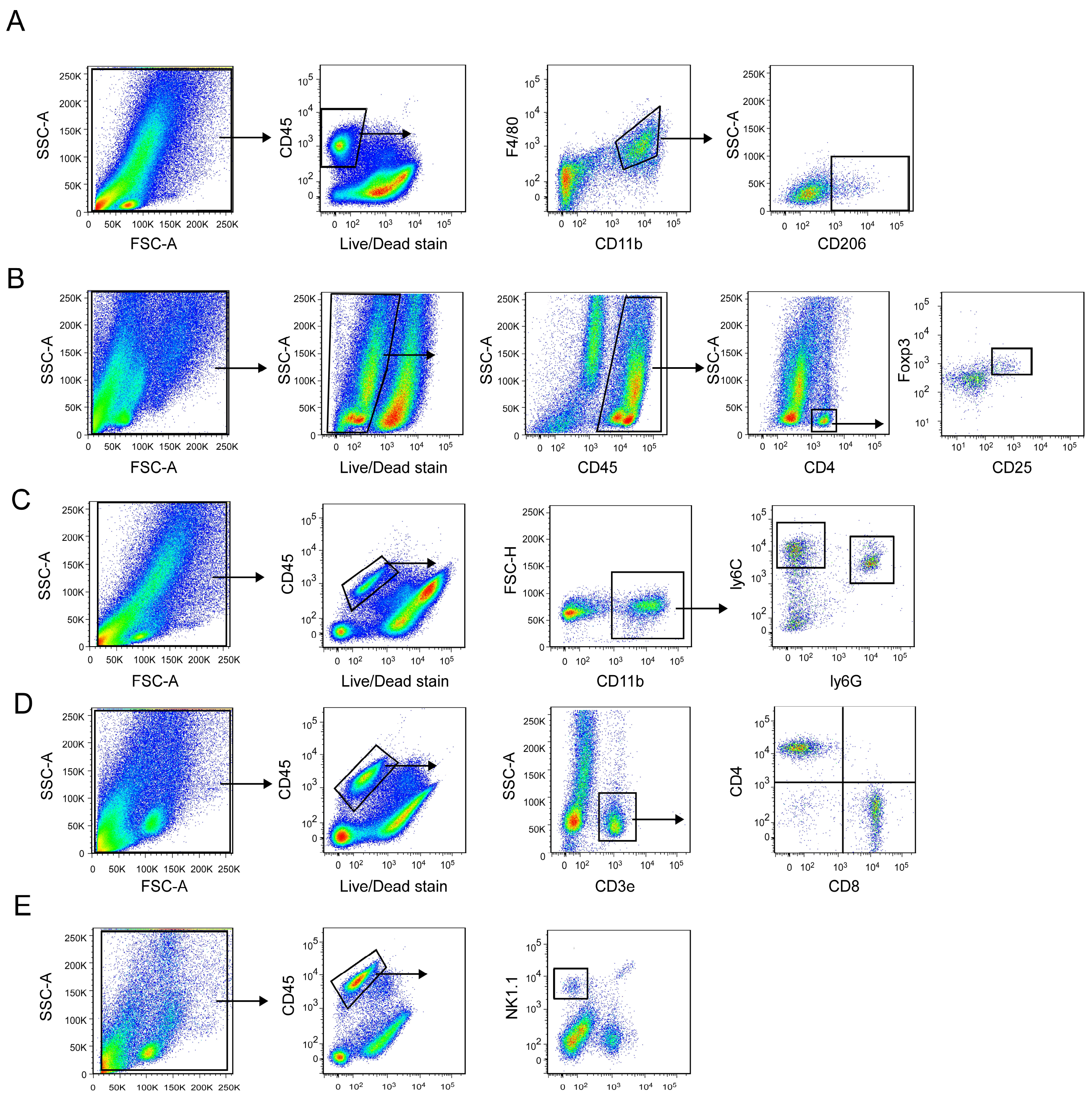

FIGURE S2 | Flow cytometry gating strategy, Related to FIGURE 2. (A) Flow cytometry gating strategy applied for identification of TAMs and M2 macrophages. (B) Flow cytometry gating strategy applied for identification of Tregs. (C) Flow cytometry gating strategy applied for identification of MDSC sub-populations. (D) Flow cytometry gating strategy applied for identification of $CD8^+$ T cells and $CD4^+$ T cells. (E) Flow cytometry gating strategy applied for identification of NK cells.

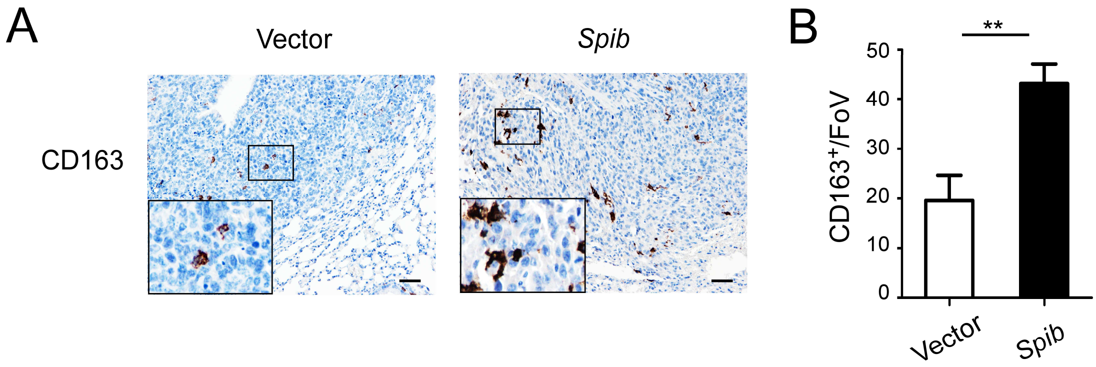

FIGURE S3 | Overexpression of *Spib* increased infiltration of M2 macrophages in the lung metastases, Related to FIGURE 2. (A) Representative pictures of CD163 staining in the lung metastases. Higher magnifications of boxed regions were displayed as insets. Scale bars, 50 μm. (B) Quantitative immunohistochemical analysis of CD163 expression in the lung metastases. FoV = field of view. n = 3 mice for each group. Mean ± SD. $^{**}P < 0.01$.

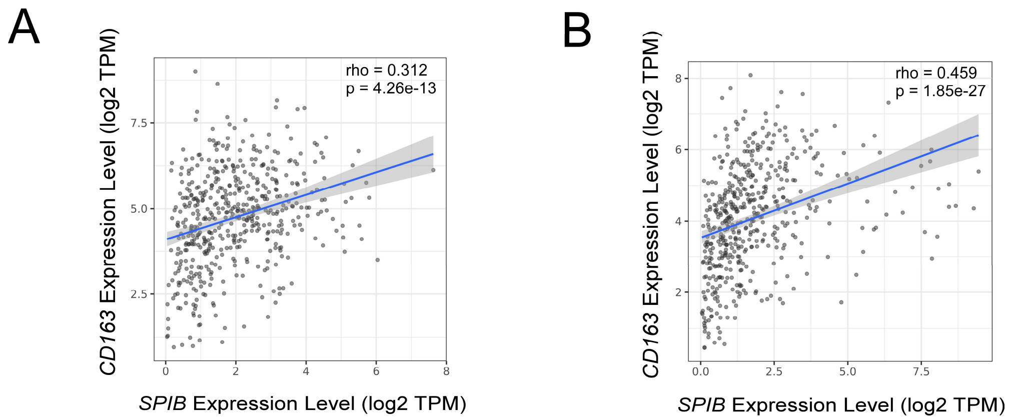

FIGURE S4 | The expression of Spi-B positively correlates with $CD163^+$ macrophages in human lung cancer tissues, Related to FIGURE 3. (A and B) The correlations between the expression of Spi-B with CD163 were shown in ADC (A) and SCC (B).
