# Supplementary material for: Spi-B Promotes the Recruitment of Tumor-Associated Macrophages via Enhancing CCL4 Expression in Lung Cancer
Source: Front Oncol. 2021 Jun 1;11:659131. doi: 10.3389/fonc.2021.659131 (PMC8205110; doi:10.3389/fonc.2021.659131)
Supplement: Supplementary file 2 [file Table_1.docx]

**Table S1: Primer sequences**

| Primers used for amplification of murine Spib |
| --- |
| Mus-Spib-ORF-F……5’ ATG GAT CCG CCA CCA TGC TTG CTC TGG AGG CT 3’ |
| Mus-Spib-ORF-R……5’ ATG GAT CCT CAG ACA TGC CGG GAG GCT G 3’ |
| Primers of shRNA targeting Spib and CCL4 |
| mSpib-shRNA-F1……5’ GAT CCC CGC AAG GTC AAA CGC AAA CTC ATT CAA GAG ATG AGT TTG CGT TTG ACC TTG CTT TTT A 3’ |
| mSpib-shRNA-R1……5’ AGC TTA AAA AGC AAG GTC AAA CGC AAA CTC ATC TCT TGA ATG AGT TTG CGT TTG ACC TTG CGG G 3’ |
| mSpib-shRNA-F2……5’ GAT CCC CGC CTC TCA AAT GCT GGG ATT ATT CAA GAG ATA ATC CCA GCA TTT GAG AGG CTT TTT A 3’ |
| mSpib-shRNA-R2……5’ AGC TTA AAA AGC CTC TCA AAT GCT GGG ATT ATC TCT TGA ATA ATC CCA GCA TTT GAG AGG CGG G 3’ |
| hCCL4-shRNA-F1……5’ GAT CCC CAT GTG CCG TGT TAT TGT ATT ATT CAA GAG ATA ATA CAA TAA CAC GGC ACA TTT TTT A 3’ |
| hCCL4-shRNA-R1……5’ AGC TTA AAA AAT GTG CCG TGT TAT TGT ATT ATC TCT TGA ATA ATA CAA TAA CAC GGC ACA TGG G 3’ |
| hCCL4-shRNA-F2……5’ GAT CCC CAG CAA GCA AGT CTG TGC TGA TTT CAA GAG AAT CAG CAC AGA CTT GCT TGC TTT TTT A 3’ |
| hCCL4-shRNA-R2……5’ AGC TTA AAA AAG CAA GCA AGT CTG TGC TGA TTC TCT TGA AAT CAG CAC AGA CTT GCT TGC TGG G 3’ |
| Primers used for RT-PCR |
| mSpib-qPCR-F……5’ GAG GTC TCG GAC AGT GAG T 3’ |
| mSpib-qPCR-R……5’ GTA CAG GCG CAG CTT CTT 3’ |
| mSpib-RT-F……5’ GCC CTT CAG TTA CCC AGA TT 3’ |
| mSpib-RT-R……5’ CGT TTG ACC TTG CGG ATT TC 3’ |
| mGapdh-F……5’ ACT CCA CTC ACG GCA AAT TCA ACG 3’ |
| mGapdh-R……5’ TCT CGT GGT TCA CAC CCA TCA CAA 3’ |
| mCsf2-F……5’ GAA GAT ATT CGA GCA GGG TCT AC 3’ |
| mCsf2-R……5’ CTT GTG TTT CAC AGT CCG TTT C 3’ |
| mIl6-F……5’ GTC TGT AGC TCA TTC TGC TCT G 3’ |
| mIl6-R……5’ GAA GGC AAC TGG ATG GAA GT 3’ |
| mCcl2-F……5’ GGA GAG CTA CAA GAG GAT CAC 3’ |
| mCcl2-R……5’ GTA TGT CTG GAC CCA TTC CTT C 3’ |
| mCcl3-F……5’ GAA GAT TCC ACG CCA ATT CAT C 3’ |
| mCcl3-R……5’ GAT CTG CCG GTT TCT CTT AGT C 3’ |
| mCcl4-F……5’ AGC TCT GTG CAA ACC TAA CC 3’ |
| mCcl4-R……5’ GGT GTA AGA GAA ACA GCA GGA A 3’ |
| mCcl5-F……5’ GAG TAT TTC TAC ACC AGC AGC A 3’ |
| mCcl5-R……5’ CCA CTT CTT CTC TGG GTT GG 3’ |
| hGAPDH-F……5’ GGT GGT CTC CTC TGA CTT CAA CA 3’ |
| hGAPDH-R……5’ GTT GCT GTA GCC AAA TTC GTT GT 3’ |
| hCSF2-F……5’ CTG CTG CTG AGA TGA ATG AAA C 3’ |
| hCSF2-R……5’ CCC TTG AGC TTG GTG AGG 3’ |
| hIL6-F……5’ GGA GAC TTG CCT GGT GAA A 3’ |
| hIL6-R……5’ CTG GCT TGT TCC TCA CTA CTC 3’ |
| hCCL2-F……5’ AGC AAG TGT CCC AAA GAA GC 3’ |
| hCCL2-R……5’ CAT GGA ATC CTG AAC CCA CT 3’ |
| hCCL3-F……5’ TGC ATC ACT TGC TGC TGA CA 3’ |
| hCCL3-R…… 5’ CTT CTG GAC CCC TCA GGC ACT 3’ |
| hCCL4-F……5’ CTC ATG CTA GTA GCT GCC TTC 3’ |
| hCCL4-R……5’ GGC TGC TGG TCT CAT AGT AAT C 3’ |
| hCCL5-F……5’ GCT GTC ATC CTC ATT GCT ACT 3’ |
| hCCL5-R……5’ CAC TTG CCA CTG GTG TAG AA 3’ |
